# Coexisting primary central nervous system non-Hodgkin’s lymphoma and colorectal adenocarcinoma: A case report

**DOI:** 10.3892/ol.2014.1818

**Published:** 2014-01-22

**Authors:** ZHENGSHI WANG, MING ZHONG

**Affiliations:** Department of General Surgery, School of Medicine, Renji Hospital, Shanghai Jiao Tong University, Shanghai 200127, P.R. China

**Keywords:** primary central nervous system non-Hodgkin’s lymphoma, colorectal adenocarcinoma, synchronous carcinomas

## Abstract

A 61-year-old female presented with night sweats following a resection for non-Hodgkin’s lymphoma of splenium corporis callosi. A positron emission tomography-computed tomography scan demonstrated that original lymphoma activity remained. A new ascending colon mass was identified simultaneously, which was diagnosed as an adenocarcinoma following the surgery. To the best of our knowledge, this is the first case to report a coexistence of primary central nervous system non-Hodgkin’s lymphoma and colorectal adenocarcinoma. The case poses a difficult clinical challenge.

## Introduction

Primary central nervous system non-Hodgkin’s lymphoma (PCNSNHL) is rare and accounts for 0.8–1.89% of intracranial tumors (1). The occurrence of PCNSNHL confounded by another malignancy is extremely uncommon. The pathogenesis of such a condition is not well established. The present study describes a case of coexisting PCNSNHL and colorectal adenocarcinoma for the first time. Written informed consent was obtained from the patient’s family.

## Case report

**Figure 1:**
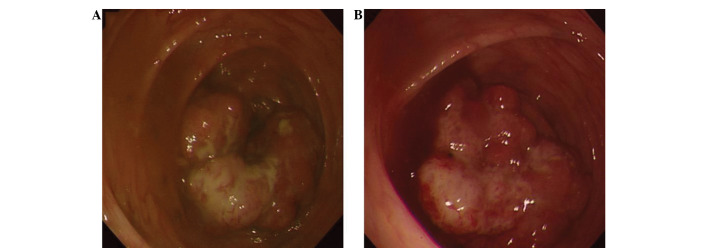
Findings under electronic colonoscope. Ascending colon mass in (A) the first colonoscopy and (B) the second colonoscopy.

**Figure 2:**
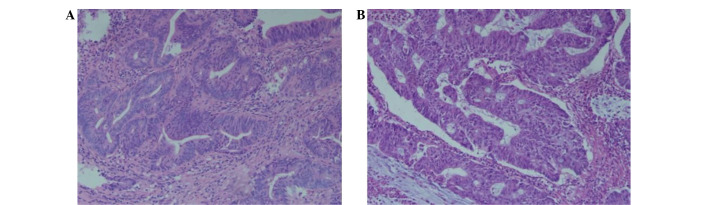
Histological appearance of ascending colon mass. (A) Hematoxylin and eosin stain of (A) high-grade intraepithelial neoplasia in the first biopsy and (B) adenocarcinoma in the second biopsy. Magnification, ×200.

A 61-year-old female was admitted to Renji Hospital (Shanghai, China) in December 2011 with a five-day history of night sweats following a resection for non-Hodgkin’s lymphoma of splenium corporis callosi. One month previously, the patient underwent contrast-enhanced cranial magnetic resonance imaging for dizziness, which indicated a splenium corporis callosi mass. Thus, the patient underwent splenium corporis callosi mass resection. Frozen pathology revealed lymphoma and immunohistochemistry identified cells that were CD19(+++), Bcl-2(+), Bcl-6(+), CD23(−), CD5 (partially positive), CD10(+), CD20(+), CD43 (partially positive), CK (pan)(−), glial fibrillary acidic protein(−), Ki-67 (60% positive), CD2 (partially positive), neuron-specific enolase (−), CD79a(+), multiple myeloma oncogene 1(+), CD3(+) and cyclin D1(+), thereby confirming the presence of B cell lymphoma. The post-operative recovery was good, but five days prior to admission, the patient experienced night sweats. Following admission, a positron emission tomography-computed tomography (PET-CT) scan was performed. The results indicated that tumor activity remained following the non-Hodgkin’s lymphoma of splenium corporis callosi resection, and an ascending colon mass was identified. Electronic colonoscopy was performed in order to identify the pathological characteristics of the ascending colon mass (Fig. 1A). Biopsy revealed an ascending colon high-grade intraepithelial neoplasia (Fig. 2A). Carcinoembryonic antigen (CEA) and α-fetoprotein serum levels were negative. Although these findings did not exclude the possibility of eventual malignancy, the patient declined further treatment for the ascending colon mass, simply accepting adjuvant chemotherapy following the lymphoma resection.

Due to the aggravating abdominal discomfort, a PET-CT scan was performed again in April 2012. The scan indicated the possibility of primary intestinal malignancy with hepatic multiple metastases and left lower lung metastasis. Biopsy under electronic colonoscope revealed an ascending colon adenocarcinoma (Figs. 1B and 2B). Therefore, hemicolectomy for the right colon carcinoma was performed. The postoperative pathological findings demonstrated a poorly and moderately differentiated ascending colon adenocarcinoma and a partial mucinous adenocarcinoma. Immunohistochemistry results were as follows: Cytokeratin 7 (CK7) (−), CK20(+), CEA(+), TOPO IIa(−), P53(−), Ki-67 (50%), HER-2(+) and cyclooxygenase 2(−).

## Discussion

Synchronous carcinomas are defined as multiple separate neoplasms that are diagnosed at the same time or within a six-month period of identifying the primary lesion. The gross and histological criteria of synchronous carcinomas, described by Warren and Gates in 1932 (2), are as follows: i) the neoplasms must be clearly malignant as determined by histological evaluation; ii) each neoplasm must be geographically separate and distinct; and iii) the possibility that the second neoplasm represents a metastasis should be excluded. Colorectal neoplasm confounded by lymphoma is in line with the criteria. The condition is uncommon. As far as we are aware, all the relevant studies are individual case reports (3–9) where the two carcinomas are localized in the same site, including the intestine or intestinal lymph nodes. Colorectal neoplasm confounded by extraintestinal lymphoma is rarer. Chang *et al* (10) reported an Epstein-Barr virus-positive case that was diagnosed as diffuse large B cell lymphoma in the cranial cavity and the ileocecal junction area. Following treatment with rituximab and chemotherapy, the cranial carcinoma disappeared, but the ileocecal lesion remained. Microscopic examination of the ileocecal lesion that was removed surgically demonstrated that it was an adenocarcinoma confounded by residual lymphoma.

**Figure 3:**
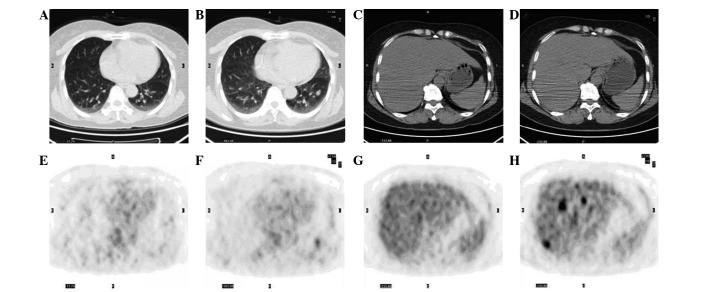
Positron emission tomography-computed tomography (PET-CT) scan images. (A and B) No pulmonary metastasis was identified in the first PET-CT scan. (C and D) Left lower lung metastasis was observed in the second PET-CT scan. (E and F) No hepatic metastasis was observed in the first PET-CT scan. (G and H) Multiple hepatic metastases were identified in the second PET-CT scan.

All the previously mentioned studies had a colorectal neoplasm and lymphoma in the same site. By contrast, the present study described the case of an elderly female with coexisting PCNSNHL and colorectal adenocarcinoma for the first time, with lymphoma in the cranial cavity and adenocarcinoma in the intestinal cavity. No hepatic or pulmonary metastases were observed in the first PET-CT scan (Fig. 3A, B, E and F), and the biopsy revealed a high-grade intraepithelial neoplasia. After four cycles of chemotherapy, hepatic and pulmonary metastases were discovered in the second PET-CT scan (Fig. 3C, D, G and H). The second biopsy revealed adenocarcinoma. Similar changes were observed in the study by Chang *et al* (10). PCNSNHL may lead to systemic immune function changes, resulting in intestinal tumorigenesis, which was accelerated by chemotherapy. Although the metastases may simply be due to chance, it is recommended that patients with PCNSNHL periodically undergo tumor marker examinations, a whole-body CT scan and electronic colonoscopy during chemotherapy.

The development of a malignancy, including colorectal neoplasm and lymphoma involves oncogenes and associated genes. The genes that are associated with colorectal neoplasm and lymphoma have been identified to include C-myc, Bcl-2 and survivin (11–17). C-myc is an oncogene that plays a central role in the genesis of numerous human cancers. Bcl-2 and survivin belong to the inhibitor of apoptosis family of proteins. These genes are likely to take part in the development of a synchronous occurrence of PCNSNHL and colorectal adenocarcinoma.

In addition, common drugs in the chemotherapy regimen for PCNSNHL are cyclophosphamide, doxorubicin, vincristine and prednisone, while those in the chemotherapy regimen for colorectal neoplasm are 5-fluorouracil, capecitabine and antitumor platinum complexes. The two groups of drugs rarely overlap with each other. Therefore, further research is required to identify how to optimize the chemotherapy regimen in patients with coexisting PCNSNHL and colorectal adenocarcinoma. C-myc, Bcl-2 and survivin may offer breakthrough treatments for this disease in the future.
